# EVASEP: A Noninterventional Study Describing the Perception of Neurologists, Patients, and Caregivers on Caregivers' Role in the Support of Patients Suffering from Multiple Sclerosis Treated with Subcutaneous Interferon Beta 1a

**DOI:** 10.1155/2016/4986073

**Published:** 2016-08-01

**Authors:** Cécile Donzé, Bruno Lenne, Anne-Sophie Jean Deleglise, Christian Kempf, Yasmine Bellili, Patrick Hautecoeur

**Affiliations:** ^1^Service de Médecine Physique et Réadaptation Fonctionnelle, Groupe Hospitalier de l'Institut Catholique de Lille, boulevard de Belfort, BP 387, 59020 Lille Cedex, France; ^2^Service de Neurologie, Groupe Hospitalier de l'Institut Catholique de Lille, boulevard de Belfort, BP 387, 59020 Lille Cedex, France; ^3^Merck Serono, 37 rue Saint Romain, 69379 Lyon Cedex 08, France; ^4^CKConsulting, 6 rue Birkenfels, 67530 Ottrott, France

## Abstract

*Background*. The perception of the role of caregivers for people with multiple sclerosis (MS) is important but poorly studied, particularly in patients with low levels of disability.* Objectives*. To describe the perceptions of the role of caregivers from the perspective of the caregiver, the patient, and neurologists.* Methods*. This observational study was conducted in France on patients with relapsing remitting MS treated with subcutaneous (SC) interferon-*β*-1a (IFN-*β*-1a) for more than 24 months.* Results*. Caregiver, patients, and neurologists all considered providing moral support and fighting against the disease as the most important role of the care provider. Moral support was considered significantly more important by caregivers than the patients and neurologists (*p* = 0.002) and caregivers considered their role in helping patients to fight disease more important than did the neurologists (*p* = 0.006). Knowledge of disease and available treatments were less important among support providers than patients (*p* = 0.007 and *p* = 0.001).* Conclusion*. There are many unmet needs in the perception of the role of caregivers for people with MS which need to be addressed to deliver the most effective care package for patients and to support the needs of the support provider.

## 1. Introduction

Multiple sclerosis (MS) is a chronic and often disabling autoimmune disease that attacks the central nervous system myelin. According to the World Health Organization and the MS International Federation (MSIF), the estimated prevalence ranges between 5 and 80 per 100,000 depending on geographical regions [1], with higher prevalence rates in developed countries, especially in Europe (80 per 100,000 in France) in 2008. According to the MSIF database the median mean age of onset of MS is 30 (range: 18.5 to 45; 31.5 in France) and the median female to male ratio is 2 (range: 1 to 7; 2.5 in France) [2].

At onset of the disease, most patients suffer from relapsing remitting MS (RRMS) [3] characterized by relapses followed by total or partial remission usually with some degree of residual disability. Relapses are followed by periods of months or even years with no new signs of disease activity. Two-thirds of the patients with RRMS will progress to secondary progressive MS. These patients have progressive neurologic decline with occasional relapses and minor remissions between acute attacks but without any more definite period of remission [4].

Treatment leads to significant decrease in the frequency of relapses, while increasing the time to disability progression and decreasing magnetic resonance imaging scan (MRI) lesion load [5–10]. However, the accrual of disability is associated with a wide range of functional deficits that can affect motor coordination, sensory functions, sphincter control, and sexual and cognitive functions. In addition to these disabling effects, fatigue and depression are common symptoms of patients with MS, meaning that patients with MS require a lot of informal support, usually provided by partners of spouses. The critical role of informal caregivers has been recognized [11–14], and it is established that daily assistance to someone with MS can be a burden [15–19] which impacts caregivers' professional and daily life [20, 21] and affects quality of life [22–24]. However, data describing the actual role of caregivers are partial and mostly reflect the point of view of the caregivers themselves rather than one of the patients or physicians. Furthermore, these assessments rarely concern early stage patients with MS with slight functional disability, even though the caregivers' perceived burden of disease may not necessarily correlate with Expanded Disability Status Scale (EDSS) score levels.

The objective of this study was to describe the role of informal caregivers according to the three main stakeholders of care of patients with MS: the patient, the caregiver, and the neurologist. Two groups of patients were recruited. The first one consisted of patients for whom a disease treatment with IFN-*β*-1a had been started one to three months prior to their recruitment, and the second one consists of patients already treated with IFN-*β*-1a for at least 24 months. The results concerning this latter group are reported here.

## 2. Methods

In accordance with the French regulation for noninterventional studies, the protocol was submitted to the French data protection authorities (Comité Consultatif sur le Traitement de l'Information en matière de Recherche (CCTIRS) and Commission Nationale de l'Informatique et des Libertés (CNIL)).

Investigators were selected by randomly phoning from a list of 485 French neurologists who were known to support patients with MS until 100 neurologists agreed to participate in the study.

Consecutively, consenting patients were included if they were between 18 and 65 years of age, were diagnosed with RRMS or secondary progressive MS with relapses, were treated with IFN-*β*-1a for more than 24 months, were able to complete the study questionnaire, and had a caregiver who also agreed to take part in the study.

The neurologist explained the scope of the study to the patient and the caregiver who both freely decided to take part in the study or not. Patients and caregivers who verbally agreed to participate were given the study questionnaire which they completed at home and then mailed it to the Contract Research Organization (CRO) in charge of the data management using prepaid envelops. The neurologist completed his/her own questionnaires and mailed them on a regular basis to the CRO. All questionnaires were independently double entered onto a database and checked for integrity, consistency, and plausibility, with requests for clarification being sent to the investigator whenever necessary.

Data were collected using three paper questionnaires, one for the patient, one for the caregiver, and one for the neurologist. Data collected by the neurologist included demographics, MS history with date of first symptoms, date of diagnosis, number of relapses over the previous two years, date of the last relapse, and EDSS score as well as current treatment of MS. The patient and the caregiver gave information on their social, family, and occupational situation and demographic data for the caregiver. They rated their understanding of the disease, their knowledge of MS treatments, their satisfaction concerning medical information provided by the neurologist, and their fear of disease progression by means of 10 cm visual analogue scales (VAS) on which 0 was the lowest score and 10 was the highest score.

The patients, caregivers, and physicians estimated the nature and frequency of assistance provided by the caregiver using a set of 12 VAS (0 = never; 10 = always). Patients and neurologists rated the physical, psychological, affective, and professional impacts of the disease (16 VAS: 0 = no impact; 10 = maximum impact) and of its treatments (15 VAS: 0 = no impact; 10 = maximum impact) on the patient, while caregivers rated these impacts on themselves (14 VAS each: 0 = no impact; 10 = maximum impact). In addition, neurologists rated the influence of the caregiver on the continuation of MS treatments (VAS: 0 = no influence; 10 = maximum influence) and described their unmet needs (open text).

Data were analysed with SAS version 9.2 (SAS Institute, Cary, NC, USA) for analyses. Analyses were performed on the data of the triads for which all three questionnaires were available. Statistical analyses were mostly descriptive and data were analysed by type of stakeholder.

The results of the triplets of stakeholders were compared by means of fixed models for repeated measures analyses of variance with the stakeholders as repeated factor.

## 3. Results

**Figure 1:**
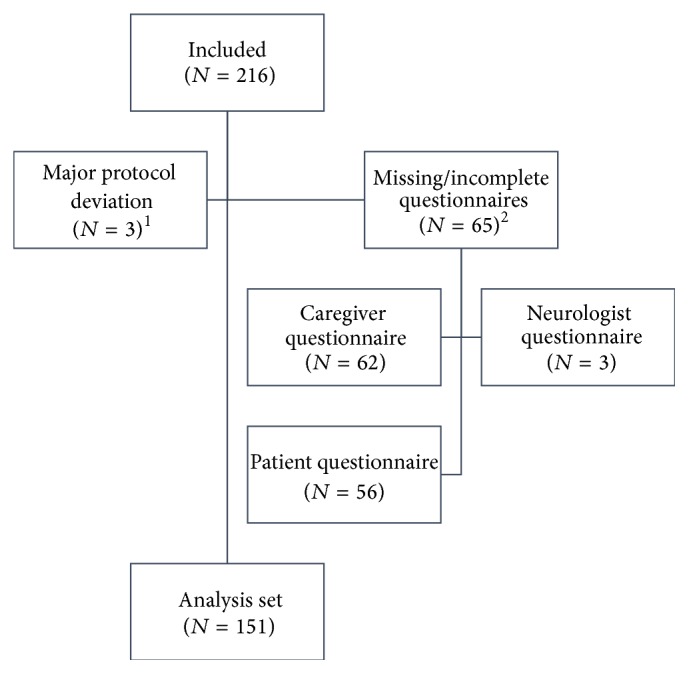
Disposition of patients. ^1^One patient had no caregiver; two patients were not treated with IFN beta 1a for more than 24 months. ^2^At least one of the three questionnaires was missing.

Between February 2008 and June 2009, 216 patients with MS were recruited to the study. For 65 patients, at least one of the three study questionnaires was missing; therefore, the analysis set consists of the 151 triplets with all questionnaires completed (see Figure 1). Descriptive statistics of the patients' and caregivers' characteristics are shown in Table 1. Patients were young (mean standard deviation (SD) age: 40.6 (9.3) years) working (66.7%) women (female/male ratio of 104/47), raising children (63.1%), living with a partner who was their caregiver in most cases (85.2%). They had been suffering from MS for 9.5 (5.6) years; the disease has been diagnosed nearly two years after the occurrence of the first symptoms (7.7 (4.9) years before inclusion) and their last relapse has occurred 2.6 (2.4) years prior to their recruitment to the study. Nearly two-thirds of the patients (64.9%) required symptomatic treatments, mostly, analgesics (42.3%) and antidepressants (19.2%).

Their caregivers were mostly working (83.9%) men (58.4%), aged 43.0 (10.9), living with a partner (92.5%) and children (61.2%). Both patients and caregivers expressed a need for more information: the level of satisfaction concerning medical information provided by the neurologist was rather low with mean (SD) scores of 6.9 (2.3) cm and 5.8 (2.5) cm for patients and caregivers, respectively, low understanding of the disease (6.5 (2.2) cm and 5.7 (2.7) cm, resp.), and low knowledge of available treatments (6.2 (2.5) cm and 6.5 (2.2) cm, resp.), while fears of disease progression were fairly high (7.4 (2.2) cm and 7.6 (2.2) cm, resp.).

**Figure 2:**
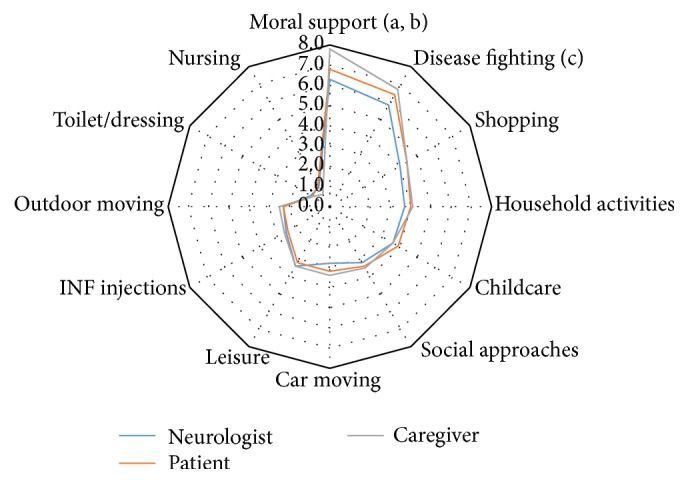
Intensity of support provided by the caregiver according to each stakeholder. ^a^Moral support: caregiver versus neurologist; *p* value < 0.0001. ^b^Caregiver versus patient; *p* value = 0.0002. ^c^Disease fighting: caregiver versus neurologist; *p* value = 0.006. *p* values are fixed model repeated ANOVA.

**Figure 3:**
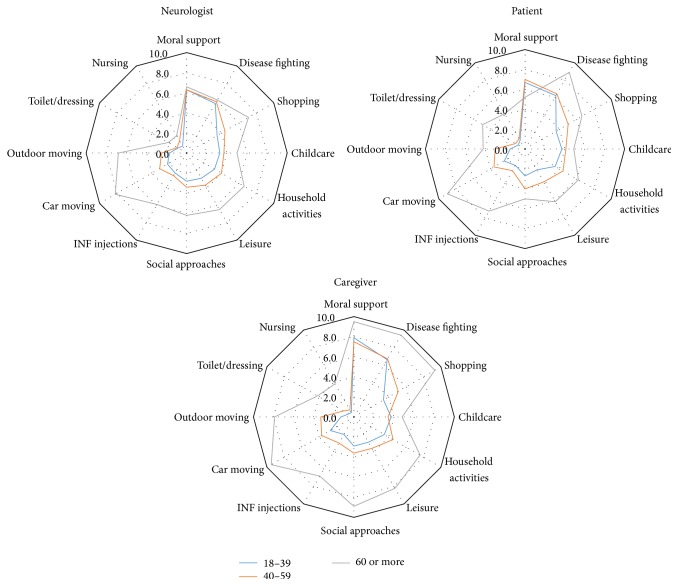
Intensity of support provided by the caregiver according to each stakeholder, by patient's age class.

**Figure 4:**
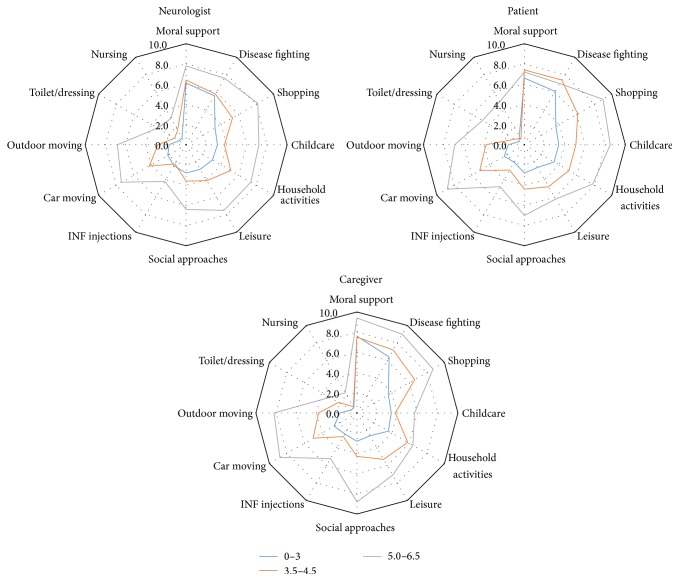
Intensity of support provided by the caregiver according to each stakeholder, by EDSS level.

**Figure 5:**
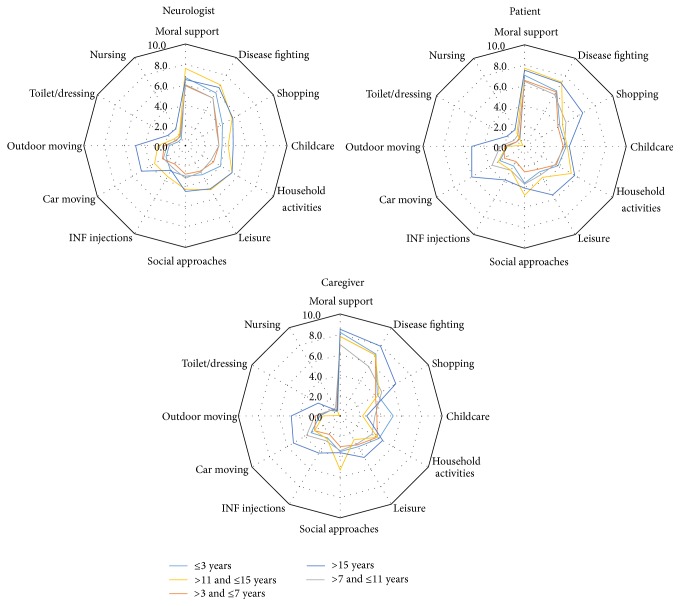
Intensity of support provided by the caregiver according to each stakeholder, by time since diagnosis.

Overall, providing moral support and fighting against the disease were regarded by all stakeholders as being the highest levels of aid provided to patients by their caregivers, while support for nursing and toilet or dressing were regarded as requiring the least support. The opinion of patients, caregivers, and neurologists on the level and kind of support provided by the caregiver was consistent for most items (see Figure 2). However, providing moral support was more significantly frequently perceived as being important by caregivers than by both neurologists (*p* < 0.0001) and patients (*p* = 0.0002). Helping to fight the disease was also perceived as being significantly more frequent by caregivers than by neurologists (*p* = 0.006). The perception of the role of the caregivers was similar for male and female patients for the three stakeholders (data not shown) but increased with the patient's age, especially according to the opinion of the caregivers themselves and with clear differences of the opinions of the three stakeholders for older patients as can be seen in Figure 3. The role of the caregiver was judged to be more important with increasing EDSS (see Figure 4), while time since diagnosis had less impact on the perceived importance of the role of the caregiver (see Figure 5).

All components of the role of the caregiver had changed with time in a similar manner for patients and caregivers (see Table 2). Disease fighting and moral support were the roles that changed most and nursing care and toilet/dressing were the least changing roles.

**Figure 6:**
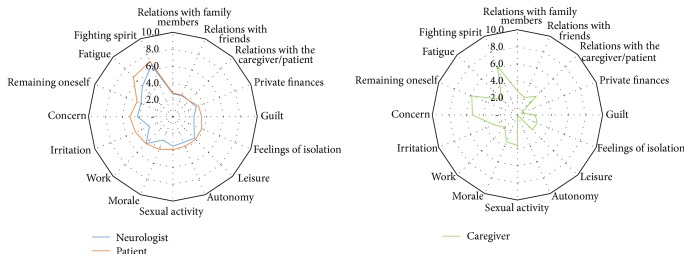
Radar plot of the physical, psychological, affective, and professional impact of the treatment. Sixteen components of the physical, psychological, affective, and professional impact of the disease on the patient were assessed by the physician and the patient, while caregivers assessed the impact of the disease on their own life. For the latter, only 14 out of 16 components were investigated: the impacts of the disease on their autonomy and finances were not considered.

Overall, the impact of the treatment on the patient was considered to be more important by the patients than by the neurologists (see Figure 6). The relative importance of the different components impacted was mostly similar for the two stakeholders. The greatest impact was on the fighting spirit of the patient, followed by fatigue, concern, irritation, and the impact on feeling of remaining oneself. The assessment of the impact of the disease on the patient work was very similar from both points of view. The impact on morale, sexual activity, autonomy, leisure, feeling of isolation, guilt, and finances appeared to be less important but still more than the impact of the disease on social relations with the caregiver, friends, or relatives.

The profile of impacts on the caregivers' life appeared to be rather different. According to caregivers, the major impacts of the disease were on their fighting spirit, on their feeling of remaining themselves, and on their concern followed by sexual activity, morale, and relations with the patient and their relatives. The impact on leisure, feeling of isolation, guilt, and relations with friends appeared to be less impacted by the disease.

The impact of the treatment on patients' life was considered to be much more important by the patients themselves than by the physician (see Figure 6). This was the case for 12 out of the 15 components investigated, with the rating of the two stakeholders being very close for the remaining three components. The hierarchy of the most affected components was similar to that described above for the impact of the disease: fighting spirit, fatigue, concern, and irritation. As was the case for the impact of the disease, relations with the caregiver, family member, and friends appeared to be less affected.

For the caregivers too, the profile of components impacted by the treatment was similar to what was described above for the impact of the disease.

**Figure 7:**
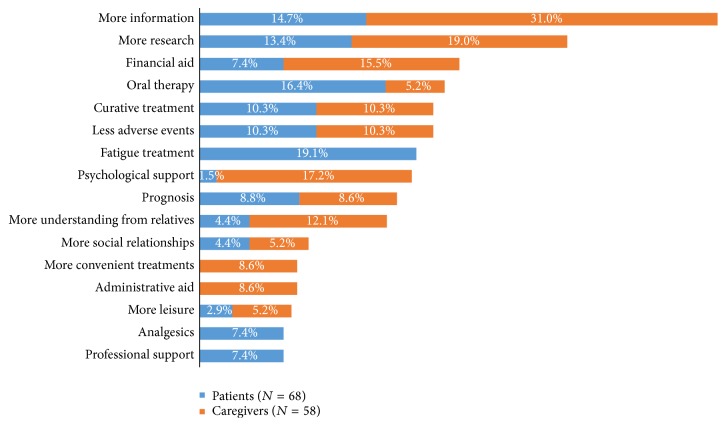
Patients' and caregivers' unmet needs.

Unmet needs were reported by 68 patients and 58 caregivers. The open text answers were classified and coded. Patients who expressed unmet needs most frequently requested not only treatments for fatigue (19.1%), oral therapy (16.4%), more information (14.7%), and more research (13.4%). For their parts, caregivers called for more information (31.0%), more research (19.0%), more psychological support (17.2%), and understanding from relatives (12.1%) but also financial aid (15.5%) (see Figure 7).

## 4. Discussion

According to patients, caregivers, and neurologists, the highest levels of implication of the caregiver in assisting patients with MS treated with interferon beta 1a for more than 24 months were for providing moral support and for fighting the disease. However, the level of implication of caregivers in providing moral support was considered to be significantly more important by caregivers than by patients or neurologists and caregivers considered their role for fighting the disease to be more important than did neurologists. The importance of the role of the caregiver was consistent for all other items assessed in our study.

Those who are expected to take an actual role of carer, typically relatives and friends, are often uncomfortable with it and it can be difficult to gain acknowledgement from them that they actually have an active role of carer [25]. At the time of diagnosis, many patients with MS are in stable relationships with their relatives and friends who are inevitably affected by the advancing disease. Partners have to cope not only with the presence of the disease but also with the unpredictability of its prognosis, which includes the possibility that their partner may become severely physically and cognitively impaired. They, therefore, face lifestyle and role adjustments that can lead to emotional distress and reduced quality of life [26]. A previous study has shown that informal caregivers with poorer mental health outcomes are more likely to provide lower-quality care and assistance and may exhibit potentially harmful caregiver behaviour [27]. Furthermore, the caregivers' feeling that providing assistance is emotionally draining or the belief that this assistance threatens their relationship with the patients significantly increases the likelihood of the caregiver's need of mental health treatment [28]. The physical, emotional, and time-intensive nature of caregiving of patients with multiple MS frequently impairs the caregiver's own physical and emotional health. Health care professionals who treat MS caregivers, or patients with MS, need therefore to be sensitive to the impact of providing assistance on emotions, relationships, and mental health needs of caregivers. They need to understand the stress, burden, and worries experienced by caregivers in order to provide appropriate mental health services and other ways of support, such as respite care, and limit the burden of caregiving. Impairment of disability, walking, and QOL of patients with MS were shown to be related to increased burden and decreased QOL of their caregivers [29]. Therefore, it may be important to provide acceptable education and support strategies with individual intervention while defining the needs and goals of the patients with MS and their caregivers to improve the rehabilitation success, even in patients with early disease and low degrees of disability.

Areas most affected by MS were similar according to patients and neurologists. They were the patient's fighting spirit, followed by fatigue, concern, irritation, and the patient's feeling of remaining oneself. For caregivers they were their fighting spirit, their feeling of remaining themselves, and their concern followed by sexual activity, morale, and relations with the patient and their relatives. This distortion in the perception of the impacts of MS for caregivers and patients can be a source of misunderstanding and communication problems between them. Both partners experience difficult feelings such as uncertainty, fear, depression, and distress. Coping with a relationship in changing situations and coping with the associated stress are best done by couples who consistently exchange their mutual perceptions, inform one another of the mental and physical strains in open discussions, and synchronise their ideas regarding the meaning and satisfaction with life. Targeted support for dyadic coping can lead to stress reduction and higher relationship satisfaction of couples living with MS.

Some invisible symptoms like fatigue and pain are prominent among patients but are rather unknown to caregivers. However, these symptoms explain part of the physical support provided at the onset of the disease even with low EDSS [16] as in our study. Buchanan et al. reported that utilisation of health providers by patients with MS increased with age [30]. In our study, the need for all components of aid increased with the disability level and consistently with age and duration of the disease, but the profiles of needs were similar for patients of either gender.

Unmet needs for patients and caregivers were different for several aspects: patients needed specific treatment for fatigue and pain and socioprofessional aids. Caregivers needed more information on the disease, psychological support, and financial support. Multiple sclerosis may cause several invisible problems related to the autonomic nervous system, cognition, mood, pain, and fatigue and caregivers may only be able to observe them partially [31]. The disease often causes unemployment from its beginning, which is likely to affect the family's finances [32]. Caregivers can be affected financially and some of them may have to limit their working time in order to support the patients. Buchanan et al. interviewed 530 caregivers of patients with MS, of which only 215 were employed. Poorer cognitive ability of the care recipient to make decisions about daily tasks and more caregiving hours per week predicted reduced caregiver employment. Better physical health domains of the caregiver quality of life were associated with significantly lower odds of reduced employment [33]. Lorefice et al. compared 497 patients with MS and 206 caregivers with a multidimensional questionnaire on how they perceived unmet needs [34]. They showed that caregivers and patients were satisfied with medical care but wanted more information on MS. Koopman et al. conducted a similar study on 353 patients with MS and 240 caregivers that showed that there was a high demand for information regarding MS and psychosocial support [31].

Our study has limitations. The study is based on results that were obtained using nonvalidated assessment tools. In order to get comparative data on the role of the three major stakeholders of MS patient care, it was necessary to actually collect data on complete triplets of stakeholders, the patient, the caregiver, and the neurologist. The study required that each patient taking part in the study had an identified caregiver who agreed to participate, and the neurologist was to check this prior to the patient's inclusion. These requirements led to a low number of patients for investigator. The noninterventional anonymous nature of the study made it impossible to send queries in case of missing data. Despite this serious limitation, it was possible to get data from the three stakeholders for nearly 70% of the patients.

## 5. Conclusions

Studies of caregivers in MS mostly concerned patients with high level of disability. Even if in our sample the disability was low (average EDSS: 2.03), we show that caregivers have an important role in supporting patients with MS.

Psychological support is the prominent part of the aid provided by caregivers.

Invisible disease symptoms like fatigue and pain are prominent among patients but not for caregivers who mostly do not identify them. These symptoms may explain at least partly the physical assistance required at the onset of the disease even in patients with low EDSS.

MS patients require important involvement of their caregivers who are in demand of information on the disease and should therefore be involved in therapeutic education programs if they wish so.

## Figures and Tables

**Table 1:** Patients' and caregivers' characteristics.

	Caregivers (*N* = 151)	Patients (*N* = 151)
Age (years)^a^	43.0 (10.9)^3^	40.6 (9.3)
18–39^b^		73 (48.3%)
40–60^b^		73 (48.3%)
>60^b^		5 (3.3%)
Gender: female^b^	62 (41.6%)^2^	104 (68.9%)
Professional activity^b^	125 (83.9%)^2^	100 (66.7%)^1^
Living in couples^b^	136 (92.5%)^4^	134 (89.9%)^2^
With children at home^b^	90 (61.2%)^4^	94 (63.1%)
Relationship	With the patient^b1^	With the caregiver^b2^
Spouse/husband	127 (84.7%)	127 (85.2%)
Child	10 (6.7%)	5 (3.4%)
Friend	5 (3.3%)	5 (3.4%)
Relative	4 (2.7%)	9 (6.0%)
Other	4 (2.7%)	3 (2.0%)
Understanding of the disease^ac^	5.7 (2.7)	6.5 (2.2)
Knowledge of available treatments^ac^	5.2 (2.8)	6.2 (2.5)
Fears of disease progression^ac^	7.6 (2.2)	7.4 (2.2)
Sufficient medical information^ac^	5.8 (2.5)	6.9 (2.3)

Time since first symptoms (years)^a4^		9.5 (5.6)
Time since diagnosis (years)^a^		7.7 (4.9)
Number of relapses in the last two years^a1^		0.9 (1.1)
Time since last relapse (years)^a2^		2.6 (2.4)
Expanded Disability Status Scale (EDSS)^*µ*^		2.0 (1–3)
0.0–3.0^b^		116 (76.8%)
3.5–4.5^b^		22 (14.6%)
5.0–6.5^b^		13 (8.6%)
Symptomatic treatments^b^		98 (64.9%)
Analgesics^b^		64 (42.3%)
Antidepressants^b^		29 (19.2%)
Sphincter disorder treatments^b^		20 (13.2%)
Antispastics^b^		18 (11.9%)
Antiasthenics^b^		10 (10.2%)

^a^Mean (SD); ^b^ *N* (%) percentages are calculated for documented values; ^c^assessed using 10 cm visual analogue scales; ^*µ*^median (quartiles).

^1,2,3,4^Number of missing values.

**Table 2:** Changes with time in the nature and frequency of the support provided by the caregiver.

	Patients (*N* = 151)	Caregivers (*N* = 151)
Car moving^a,§^	3.1 (3.5)^4^	3.1 (3.3)^5^
Outdoor moving^a,§^	2.5 (3.1)^4^	2.7 (3.2)^3^
Household activities^a,§^	3.4 (3.2)^1^	3.8 (3.5)^4^
Shopping^a,§^	3.5 (3.5)^6^	3.7 (3.5)^2^
Childcare^a,§^	2.9 (3.1)^28^	2.9 (3.2)^29^
Leisure^a,§^	2.9 (2.8)^5^	3.2 (3.1)^5^
Disease fighting^a,§^	5.0 (3.3)^3^	5.0 (3.2)^7^
Moral support^a,§^	4.7 (3.2)^3^	5.1 (3.4)^2^
INF injections^a,§^	2.5 (3.3)^3^	2.7 (3.3)^8^
Nursing^a,§^	1.4 (2.3)^19^	1.0 (1.9)^16^
Social approaches^a,§^	2.6 (3.2)^5^	2.7 (3.4)^7^
Toilet/dressing^a,§^	1.3 (2.2)^8^	1.1 (2.1)^8^

^a^Mean (SD); ^§^assessed using 10 cm visual analogue scales.

^1,2,3,4,5,6,7,8,16,19,28,29^Number of missing values.

## Abbreviations

MS: Multiple sclerosis
VAS: Visual analogue scales
WHO: World Health Organization
MSIF: Multiple Sclerosis International Federation
RRMS: Relapsing remitting multiple sclerosis
IFN: Interferon
MRI: Magnetic resonance imaging scan
EDSS: Expanded Disability Status Scale
CCTIRS/CNIL: French data protection authorities
CRO: Contract Research Organization
SD: Standard deviation.
